# Implications of the Gut Microbiome in Alzheimer’s Disease: A Narrative Review

**DOI:** 10.7759/cureus.73681

**Published:** 2024-11-14

**Authors:** Palvi Mroke, Raman Goit, Muhammad Rizwan, Saba Tariq, Abdul Wahid Rizwan, Muhammad Umer, Fariha F Nassar, Angela Juliet Torijano Sarria, Dilpreet Singh, Imran Baig

**Affiliations:** 1 Internal Medicine, Caribbean Medical University School of Medicine, Willemstad, CUW; 2 Internal Medicine, Virgen Milagrosa University Foundation, San Carlos City, PHL; 3 Internal Medicine, Sheikh Zayed Medical College, Rahim Yar Khan, PAK; 4 Internal Medicine, Amna Inayat Medical College Pakistan, Lahore, PAK; 5 Internal Medicine, King Edward Medical University, Lahore, PAK; 6 Internal Medicine, Rajiv Gandhi University of Health Science, Bangalore, IND; 7 General Practice, Universidad Santiago de Cali, Cali, COL; 8 Internal Medicine, Ascension St. John Hospital, Detroit, USA; 9 Internal Medicine, Houston Methodist Hospital, Houston, USA

**Keywords:** alzheimer's disease, amyloid cascade hypothesis, amyloid plaques, dysbiosis, gut-brain axis, gut microbiome, gut microbiome and mental health, neuroinflammation and mental health, tau hypothesis, tau-protein

## Abstract

Alzheimer’s disease (AD) is a progressive neurodegenerative disorder, with its prevalence doubling approximately every decade. It is a significant contributor to disability-adjusted life-years in individuals aged 50 and older, impacting a substantial portion of this population globally. The pathophysiology of AD is primarily explained by two hypotheses: the amyloid cascade hypothesis and the tau hypothesis. While the amyloid cascade hypothesis is widely accepted as the main contributor to AD, both mechanisms promote neuroinflammation by driving the formation of amyloid-beta (Aβ) plaques and tau tangles, which are key features of the neurodegenerative process. Recent studies highlight the critical role of the gut microbiome (GMB) in the progression of AD. Gut dysbiosis has been linked to neuroinflammation, altered Aβ metabolism, blood-brain barrier disruption, and changes in neuroactive metabolites. Targeting the GMB offers potential therapeutic avenues aimed at restoring microbial balance and mitigating the effects of dysbiosis. The gut-brain axis, crucial for neurological health, remains underexplored in AD, especially since current research is limited to animal models and small human studies, leaving uncertainty about specific gut bacteria’s roles in AD. Currently, pharmacological treatments for AD include cholinesterase inhibitors and memantine. This review discusses newer and emerging treatments targeting Aβ and tau pathology, alongside microbiome-based interventions. Larger, human-based studies with diverse populations are essential to establish the therapeutic efficacy of these microbiome-targeted treatments and their long-term impact on AD management.

## Introduction and background

Defined by Alois Alzheimer in 1906, Alzheimer’s disease (AD) is known as the insidious onset and progressive behavioral and cognitive decline mostly seen in individuals aged 65 and older [1,2]. This neurodegenerative disease is counted as the fourth leading cause of disability-adjusted life-years lost in those aged 75 and older and is globally recorded as affecting 22% of all persons aged 50 and above; however, the disease prevalence doubles every 10 years [2,3]. The staging classification of the disease is outlined in the Diagnostic and Statistical Manual of Mental Disorders, 5th edition and is distinguished by the degree of behavioral and cognitive impairment [1]. It can be categorized as preclinical or presymptomatic, mild cognitive impairment, and mild, moderate, and severe dementia stages [1]. The most common clinical presentation observed is episodic short-term memory loss, specifically retaining the ability to recall long-term memories while facing difficulty retaining new ones [1]. As the disease progresses, additional language, visuospatial skills, and higher executive functioning, along with neuropsychiatric symptoms such as disinhibition, agitation, psychosis, and wandering, are exhibited [1,2].

Although clinical presentation is usually sufficient for the initial diagnosis of AD, CSF biomarkers amyloid-beta 42 (Aβ42), phosphorylated tau (p-tau), and total tau are confirmatory tools [1]. Two commonly known pathophysiological mechanisms for AD are the amyloid cascade hypothesis and the tau hypothesis, with the amyloid hypothesis being the most widely accepted [1]. AD is associated with the accumulation of both the extracellular beta-amyloid protein fragment (also known as β-amyloid (Aβ) plaques) and intraneuronal tau protein (also known as tau tangles) [4,5]. Although it remains unclear which pathophysiological mechanism initiates the neurodegenerative process of AD, researchers suspect the incipient process is Aβ plaque deposition followed by tau protein depositions [5]. Additionally, early-onset familial AD is linked to amyloid precursor protein (APP) mutation and senile Aβ plaques, further supporting the hypothesis of Aβ deposition as the initiating pathological event in AD [6]. However, recent evidence does challenge that Aβ deposition in senile plaques is a late, nonspecific event, therefore proposing that tau phosphorylation and aggregation are the favorable cause of neuroimmunomodulation decline in AD [7].

Both hypotheses ultimately result in neuroinflammation, a central feature of AD, which is regulated by the trillions of human bacteria, archaea, protozoa, viruses, and fungi, combined and referred to as the gut microbiome (GMB) [8]. CNS immune response alteration in the context of AD can be a result of the alterations made in the microbial-derived metabolites and peripheral immunity via GBM-mediated changes. Although the precise mechanism remains elucidated, recent studies indicate that the potential alteration of GMB is associated with AD compared to those without AD [8,9]. This narrative review aims to explore and summarize the evidence regarding the implications of the GMB on AD.

## Review

Composition and function of the GMB

**Figure 1:**
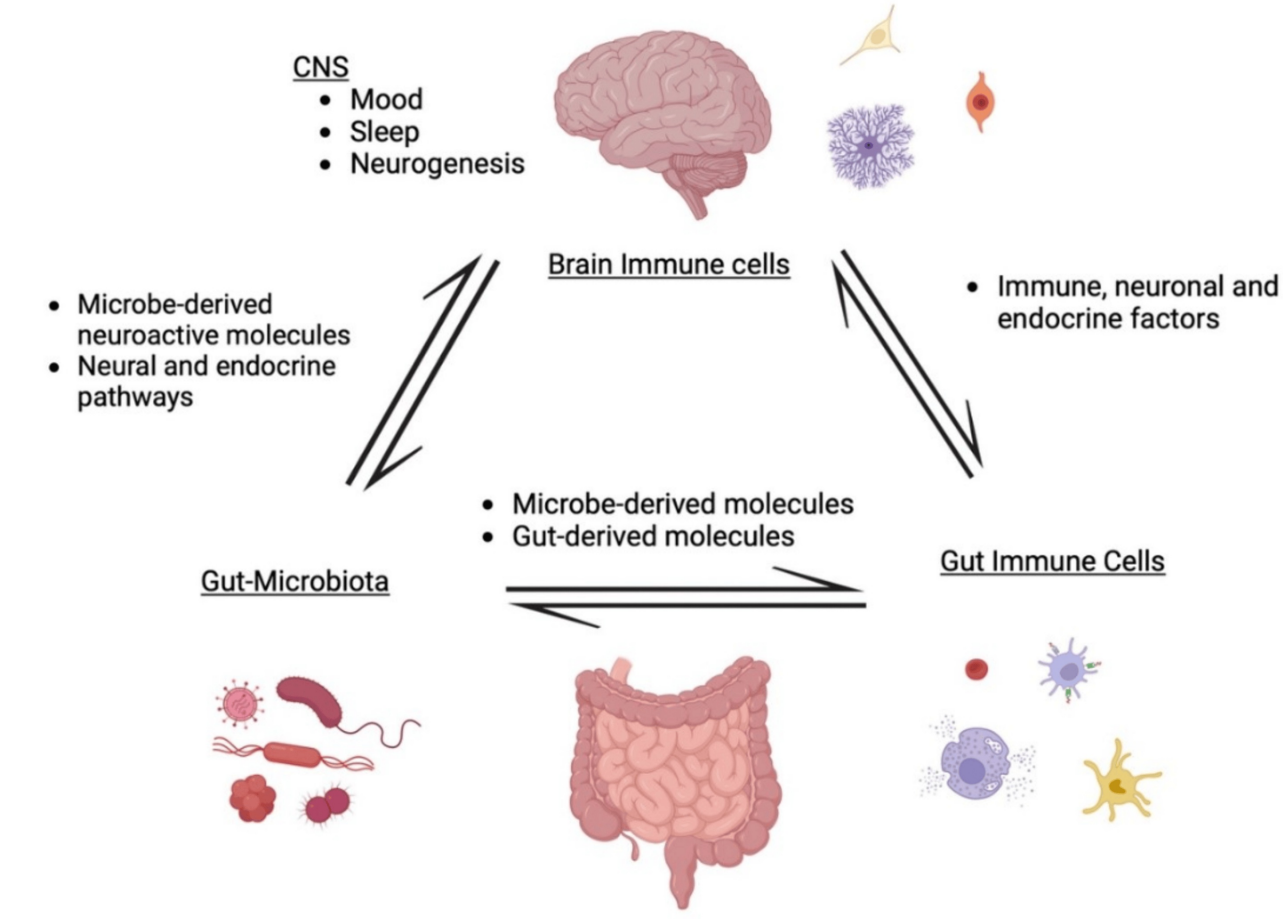
Bidirectional communication between CNS and gut interaction of gut microbiota The biodirectional communication produces neuroactive molecules that influence brain function and the release of gut-derived molecules, such as neurotransmitters and hormones, which signal to the brain. The brain, in turn, can influence gut function through the ANS. This communication network, involving both neural and immune pathways, is essential for maintaining overall health and well-being. ANS: autonomic nervous system Figure created with BioRender; image credit: Muhammad Rizwan

The human gastrointestinal tract represents an immense interface that encounters a plethora of environmental factors and antigens; annually, 60 tons of food is passed through the tract, comprising a wide variety of bacteria, archaea, and eukaryotes that collectively constitute the gut microbiota. This gut microbiota has coevolved over time with the host, adapting to the physiologic responses and dietary patterns. Through this, the microbiota developed specialized functions like supporting digestion and modulating immune responses, and the host developed intestinal barriers and immune tolerance [10]. It provides physiological and immunological functions like strengthening gut integrity, harvesting energy, and protecting against pathogens [11]. Gut microbiota consists of Firmicutes, Bacteroidetes, Actinobacteria, Proteobacteria, Fusobacteria, and Verrucomicrobia, with Firmicutes and Bacteroidetes representing 90% of gut microbiota, with Clostridium genera accounting for 95% of Firmicutes phyla [12]. The estimated cell count for the makeup is more than 10 trillion cells, with hundreds to thousands of microbial species in everyone. There are more genes in the microbiome than in the human genome. The gut microbiota is capable of converting host-derived biochemical molecules, interfering with endocrine and metabolic processes (Figure 1), and activity of therapeutic drugs [13]. For example, one recent research study showed that gut microbiota modulates xenobiotic metabolism through a variety of mechanisms involving the re-activation of otherwise inactive drug metabolites, immune cell dynamics, and alteration in the level of enzymes in the gut and liver. This can alter the quality and toxicity of the medicine, which in turn can cause clinical derangements and confusion with other diseases that also alter the enzymes in the gut and liver [14-17].

Gut-brain axis

The gut-brain communication is mediated by neural, immune, and endocrine pathways, with gut microbes and their metabolites playing a crucial role in neurological health. Enteroendocrine cells in the gut epithelium [18] secrete over 20 signaling molecules influenced by microbial metabolites like short-chain fatty acids (SCFAs) and bile acids. They enter the systemic circulation and affect CNS functions, including ingestive behavior. Dietary intake significantly regulates these microbial activities. SCFAs are key mediators produced from dietary fiber, activating L cells to release peptides such as GLP-1, which controls satiety and behavior [19,20]. Immune pathways also play a critical role; in a healthy gut, immune responses shift toward anti-inflammatory actions, mediated by regulatory T cells. Therefore, dysbiosis can lead to local and systemic inflammation, affecting the CNS through immune cell activation and release of inflammatory mediators, highlighting the bidirectional communication between the gut and brain [21]. Disruptions of this gut microbiota are linked to disorders such as autism spectrum disorder, Parkinson’s disease, AD, depression, and anxiety. Alterations in microbial diversity can negatively impact the CNS, influence brain function, and contribute to the development and progression of these neuropsychiatric conditions [22,23].

Pathophysiology 

The GMB is a dynamic and complex ecosystem comprising trillions of microorganisms, including bacteria, viruses, fungi, and archaea, that reside in the human gastrointestinal tract. This microbial community plays an essential role in various physiological processes, including digestion, metabolism, immune regulation, and the maintenance of intestinal barrier integrity [24,25]. The pathophysiology of AD is complex and multifactorial, involving genetic, environmental, and lifestyle factors [26]. The GMB represents a novel and increasingly recognized contributor to this complexity, through mechanisms such as neuroinflammation, modulation of Aβ metabolism, disruption of blood-brain barrier (BBB) integrity, and the production of neuroactive metabolites [26]. The GMB may play a critical role in the development and progression of AD [27-29]. Understanding these mechanisms opens new avenues for potential therapeutic interventions targeting it [30]. The concept of the gut-brain axis has introduced the idea that the GMB also significantly impacts brain function and health. Emerging evidence suggests that dysbiosis, or an imbalance in the gut microbiota, may contribute to the development and progression of neurodegenerative diseases, particularly AD [30,31].

AD is characterized by the accumulation of Aβ plaques and tau protein tangles in the brain, leading to neuroinflammation, synaptic dysfunction, and neuronal loss. While the exact etiology of AD is not fully understood, a growing body of research implicates GMB as a potential modulator of the disease’s pathogenesis [31-33]. Neuroinflammation is a central feature of AD and is thought to play a key role in its pathogenesis. The GMB is closely linked to the immune system and can influence inflammatory responses in the body. Dysbiosis can lead to the disruption of the intestinal barrier, resulting in increased intestinal permeability, often referred to as “leaky gut.” This allows bacterial endotoxins, such as lipopolysaccharides (LPS), to translocate into the bloodstream. Once in circulation, LPS can cross the BBB and trigger an inflammatory response in the brain by activating microglia, the brain’s resident immune cells [8,27,32-35].

**Figure 2:**
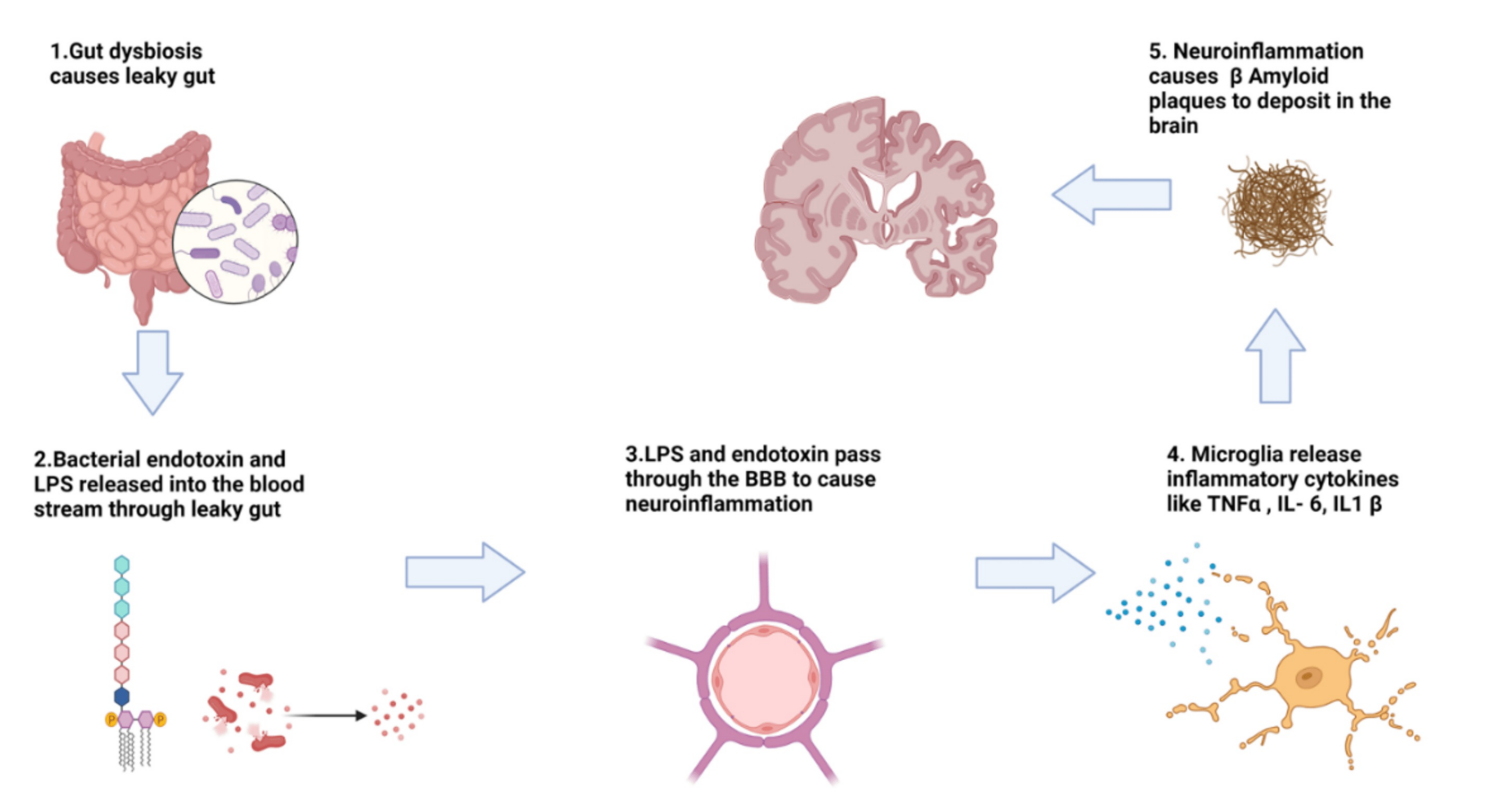
Gut dysbiosis mechanism in AD The gut dysbiosis mechanism allows bacterial endotoxins and LPS to enter the bloodstream, thereby resulting in a leaky gut. These harmful molecules cross the BBB, activating microglia in the brain. In response, microglia release inflammatory cytokines, which trigger the deposition of Aβ plaques, contributing to the development of AD. Aβ: amyloid-beta; AD: Alzheimer’s disease; BBB: blood-brain barrier; LPS: lipopolysaccharides; TNFα: tumor necrosis factor alpha Figure created with BioRender; image credit: Fariha F. Nassar

Activated microglia then release pro-inflammatory cytokines such as IL-1β, tumor necrosis factor alpha (TNFα), and IL-6, which can exacerbate neuronal damage and contribute to the progression of AD [36]. Chronic neuroinflammation is thought to promote the formation of Aβ plaques and tau tangles, further driving the neurodegenerative process. Therefore, gut dysbiosis-induced systemic inflammation and microglial activation represent a significant pathway through which the GMB may contribute to AD pathology (Figure 2) [25].

Aβ is a peptide that aggregates to form plaques, one of the hallmark features of AD. The production and clearance of Aβ are tightly regulated processes, and any imbalance in these processes can lead to its accumulation in the brain. The GMB may influence Aβ metabolism through several mechanisms [26]. Certain gut bacteria can produce amyloid-like proteins that may stimulate cross-seeding of Aβ in the brain, promoting its aggregation. Additionally, the GMB can modulate the activity of enzymes involved in Aβ production, such as β-secretase (BACE1) and γ-secretase, through the production of microbial metabolites. For example, SCFAs produced by gut bacteria have been shown to influence Aβ processing. While SCFAs have neuroprotective effects at physiological levels, dysbiosis can alter their production, potentially leading to changes in Aβ metabolism that favor plaque formation [8,24,27].

Furthermore, the GMB can affect the clearance of Aβ. The glymphatic system, which is involved in the clearance of waste products from the brain, including Aβ, can be influenced by systemic inflammation and immune responses originating from the gut. Dysbiosis-induced neuroinflammation may impair glymphatic clearance, contributing to the accumulation of Aβ in the brain [24,26]. This connection extends to its impact on the BBB; the BBB is a selective permeability barrier that protects the brain from potentially harmful substances in the blood while allowing the passage of essential nutrients and signaling molecules [8]. The integrity of the BBB is crucial for maintaining a stable environment for neuronal function, as dysbiosis has been shown to compromise BBB integrity through several mechanisms [25,27]. Firstly, the production of LPS by gram-negative bacteria in the gut can lead to systemic inflammation, which may weaken the BBB [25,33,34]. Inflammatory cytokines, such as TNFα, can disrupt tight junctions between endothelial cells in the BBB, increasing its permeability [25,26]. This allows harmful substances, including LPS and pro-inflammatory cytokines, to enter the brain, where they can induce neuroinflammation and contribute to AD pathology [8]. Secondly, gut-derived metabolites, such as SCFAs, play a role in maintaining BBB integrity. SCFAs, particularly butyrate, are known to strengthen tight junctions in the BBB. Dysbiosis, which can result in reduced SCFA production, may therefore compromise BBB function and allow for the infiltration of neurotoxic substances into the brain [28,35,37].

The GMB produces a variety of metabolites that can influence brain function and health. These include SCFAs, neurotransmitter precursors, and bile acids. Dysbiosis can alter the production of these metabolites, potentially contributing to the pathogenesis of AD. SCFAs, such as acetate, propionate, and butyrate, have been shown to exert neuroprotective effects by modulating immune responses, enhancing BBB integrity, and promoting the production of brain-derived neurotrophic factor (BDNF), a protein involved in synaptic plasticity and neuronal survival. Dysbiosis-induced reductions in SCFA production may, therefore, impair these protective mechanisms, contributing to AD progression [8,25,27].

Moreover, the GMB can influence the synthesis of neurotransmitters such as serotonin and gamma-aminobutyric acid, which play critical roles in mood regulation and cognitive function. Dysbiosis may lead to altered levels of these neurotransmitters, potentially contributing to the cognitive and behavioral symptoms observed in AD [28]. Additionally, bile acids, which are metabolized by gut bacteria, have been implicated in the regulation of neuroinflammation and Aβ metabolism. It can lead to changes in bile acid composition, which may influence AD pathology through effects on the brain’s immune response and Aβ clearance [25,36-38].

Therapeutic interventions 

The treatment modality in patients with AD patients involves alleviating symptoms, halting the progression of neurodegeneration, and preserving cognitive function [39]. Currently, two classes of pharmacological therapy are approved for the management of AD, which include cholinesterase inhibitors and memantine [40]. Newer therapeutic interventions under investigation target the overproduction of Aβ42 using γ-secretase inhibitors, β-secretase inhibitors, or α-secretase enhancers; decrease Aβ accumulation in senile plaques through aggregation inhibitors; and enhance Aβ clearance via active or passive immunotherapy. Other novel approaches like focused ultrasound with microbubbles have shown promising results in animal models, but more research is needed to apply to humans; there is a need to consider that this requires advanced ultrasound training by physicians for its implementation [41-43]. Additionally, there are treatments targeting tau pathology, including drugs that inhibit the aggregation of p-tau, such as leuco-methylthioninium bis(hydromethanesulfonate), a methylene blue derivative; drugs targeting glycogen synthase kinase 3 that reduce tau phosphorylation; and immunotherapies that elicit an immune response against hyperphosphorylated tau protein [39].

As we previously pointed out, GMB plays an important role in AD, and therapeutics for AD impacting GMB include probiotics, prebiotics, postbiotics, synbiotics, and fecal microbiota transplantation (FMT). Among these, probiotics have been extensively studied in human clinical trials, with encouraging outcomes. Recent evidence from 27 animal and 11 human trials showed that probiotics had a significant positive effect on slowing cognitive decline in patients with AD [44].

Several mechanisms are behind the pathogenesis of AD, of which neurotrophic factors, oxidative stress, and inflammation play major roles [45]. It is found that BDNF, a neurotrophic protein, has a protective role in degenerating neurons in AD, and patients with AD exhibit significantly lower serum BDNF levels compared to healthy individuals, particularly in the later stages of the disease [46]. Evidence highlights that probiotics, particularly *Lactobacillus plantarum *DW2009, have several beneficial roles in preserving and halting the decline of cognitive function among patients with AD. They have a downregulatory effect on inflammatory factors like IL-1β, leading to an upregulation of BDNF. Hence, early intervention with Lactobacillus plantarum supplement helps alleviate mild cognitive impairment, highlighting the importance of the gut-brain axis [47,48]. Oxidative stress plays a key role in AD by promoting Aβ accumulation, altering neuronal lipids, increasing harmful byproducts like 4-hydroxynonenal and malondialdehyde, modifying proteins, and increasing levels of lipid peroxidation and protein carbonyls. Probiotics may help counteract this by boosting antioxidant enzymes like superoxide dismutase, effectively reducing oxidative damage and its effects on AD progression [47]. There is strong research that investigated the microbiome of multispecies in probiotics containing *Lactobacillus acidophilus*, *Lactobacillus casei*, *Bifidobacterium bifidum*, *Lactiplantibacillus plantarum*, and *Lactobacillus fermentum *that highlighted their potentials as probiotics supplements in cognitive improvement among patients with AD. However, these studies were carried out over a short duration of approximately 12 weeks; this time limitation poses challenges in fully understanding the long-term impact of probiotics as AD disease progresses over years. The short duration may not capture sustained or cumulative effects, and variations in individual responses might not be fully representative [29,48,49]. Nonetheless, recent studies conducted within the past decade on microbiome and probiotics showed promising evidence of cognitive improvement in patients with AD when given in the early stages of the disease.

Dietary interventions 

The GMB produces metabolites in association with the intestinal mucosa, thus maintaining health and homeostasis [50,51]. The gut microbiota is dependent on food and may be altered depending on an individual's particular diet. To illustrate, fermentation of a high-fiber diet in the gut releases metabolites that help regulate colonic epithelium proliferation [50]; these metabolites enter the systemic circulation and induce beneficial immunomodulatory effects on other organs of the body. This explains the correlation between a high-fiber diet and the reduction in risk of various cancers, cardiovascular diseases, obesity, and diabetes mellitus [52,53]. In some animal studies, deficiency of dietary fiber has been shown to cause cognitive impairment and memory loss and negatively impact activities of daily living [54]. These studies further demonstrated structural changes in the hippocampus and disturbances in the gut microbiota, which may be associated with cognitive decline [54]. Dietary factors have also been shown to influence the risk of AD [55,56]. Deficiency of antioxidants in the diet, like vitamins E and C, and vitamins B9, B6, and B12, may play a role in disease development. Antioxidants reduce Aβ-induced lipid peroxidation and oxidative stress, thus suppressing inflammation [55,57,58]. In addition, vitamin D and minerals like calcium and magnesium in recommended amounts have a beneficial effect on AD patients. A high-fat diet and excess saturated fatty acids promote hyperinsulinemia, inflammation, and hypercholesterolemia, which may cause oxysterols to accumulate in the brain of Alzheimer’s patients, worsening disease progression [57]. A high carbohydrate diet also seems to affect cognitive ability and may play a role in mild cognitive impairment; however, carbohydrates in the form of dietary fiber are an exception.

The Mediterranean diet affects the gut microbiota and is associated with less cognitive decline in patients with mild cognitive impairment or stroke [59]. Moreover, the Dietary Approaches to Stop Hypertension (DASH) diet has been shown to have a neuroprotective effect in improving cognitive deficits, memory, and spatial learning. The role of the Mediterranean plus DASH intervention for the neurodegenerative delay (MIND) diet was studied and showed significant neuroprotective effects comparable to those observed with the DASH-only diet in the group that had high adherence to the MIND diet [59]. These studies show a promising role of diet and GMB in enhancing cognition and overall brain health. This role should be investigated further with a larger sample size and may aid in developing specific diet interventions to prevent or slow the progression of AD.

FMT

FMT involves transferring feces from a healthy donor into the GI tract of a recipient for therapeutic purposes such as the management of clostridium difficile infection, inflammatory bowel disease, metabolic syndrome, autoimmune disease, and neurological disorders [60-62]. Recent clinical trials performed on mice have shown the benefit of FMT in controlling symptoms of AD. Compared to prebiotics, probiotics regulate gut microbes and improve cognitive impairment [61]. In one study, the transplantation of fecal microbiota from wild-type mice into transgenic model mice expressing APP, presenilin-1, and microtubule-associated protein tau transgenes showed a reduction in the formation of Aβ plaques, neurofibrillary tangles, glial reactivity, and cognitive impairment. Additionally, this study included several case reports providing evidence of FMT use in Clostridium difficile patients with AD, showing successful treatment of the infection and improvements in cognitive decline [24]. FMT is delivered either by upper or lower GI routes. Upper GI route includes esophagogastroduodenoscopy, nasogastric, nasojejunal, or nasoduodenal tube. The lower GI route involves colonoscopy, retention enema, and oral capsule [63,64]. Patient preparation for successful FMT involves standard screening protocols, recipient education, and antibiotic restriction 12-24 hours before fecal infusion [60,61]. The recipient needs bowel lavage regardless of upper or lower GI routes [64]. The bowel should be free of contaminated fecal material before the donor feces infusion. Some studies suggested the use of loperamide one hour before FMT to ensure that the transplanted feces stay at least four hours long in the intestines [65]. Regardless, there is insufficient evidence to showcase a superior route for FMT. The route of delivery should be based on an individual patient’s situation [61]. While promising in animal models, large-scale human trials are needed to confirm FMT’s role in managing AD and to establish standardized clinical protocols.

There is considerable evidence suggesting that FMT may have a role in the prevention and treatment of AD. A recent study concluded that mice treated with FMT demonstrated better spatial learning ability and memory compared to the non-FMT-treated mice [61,66]. This study showed the neuroprotective effects of FMT against AD in APPswe/PS1dE9 transgenic mice, which included improvement in cognitive deficits, decrease in neuroinflammation, and amyloid beta accumulation [66]. The gut microbiota in AD patients notably differs from that within healthy patients; thus, AD patients may not be able to metabolize certain peptides and inflammatory mediators [67]. In healthy patients, the gut bacteria produce tryptophan and SCFAs during metabolism, which decrease inflammation. FMT-treated mice restore the SCFA, which disrupts amyloid beta oligomers, thus halting AD disease progression and contributing to improved cognition [67]. These studies in animals show a promising role of FMT for prevention and management of AD; however, ethical obligations have limited studies in humans. [63]. We advise conducting additional investigations on humans regarding the role of FMT in regulating cognition, on a larger scale, keeping the ethical obligations in mind.

Emerging technologies and approaches

Recent avant-garde microbiome research has acknowledged a significant connection between AD and gut microbes. Metagenomics, metabolomics, and bioinformatics are the innovative technologies that have refined our understanding of how the GMB impacts the neurodegenerative system. These technologies provide intuition into how gut dysbiosis may contribute to AD pathogenesis through the comprehensive analysis of microbiomes and their metabolic products [28]. The metagenomic technique is a rapidly developing technology that works by sequencing the collective DNA of gut microbes, which researchers can use to identify and characterize the microorganisms present; furthermore, determining their relative affluence and their functional capabilities. It helps in the identification of microbe metabolites, such as LPS and SCFAS, that potentially influence brain health. In the context of AD, metagenomics has been instrumental in identifying specific gut microbiota that may be associated with the disease [8]. Metabolomics is a rising technology that allows a comprehensive analysis of small metabolites produced by microbes and their interaction with the host catabolism. Metabolomics has uncovered changes in the levels of harmful metabolites like LPS in AD patients. Raised LPS levels can lead to systemic inflammation and may worsen AD pathology by enhancing Aβ aggregation and tau phosphorylation [68]. These findings emphasize the potential of metabolomics in identifying new biomarkers for AD and developing microbiome-based therapeutic strategies. Bioinformatics is a technique that involves computerized technology to collect, store, analyze, and display biological data and information, such as DNA and amino acid sequences. Bioinformatics helps researchers identify the specific microbial agent and their metabolites by analyzing DNA sequences that may lead to the progression of AD.

The idea of using personalized microbiome-based therapies for Alzheimer’s is another appealing area of research. It combines our understanding of gut health with brain health, targeting to create customized treatments that could help people with Alzheimer’s live better lives. The GMB is highly divergent, with significant variability across individuals, making it challenging to draw consistent conclusions from bioinformatics analyses. This complexity can lead to difficulties in replicating findings across different studies, and integrating multi-omics data is complex and difficult to interpret [69]. For this reason, physicians need to focus on grasping a better understanding of the basics and the needs of the current era [70].

Research gaps and future studies

There are still several unanswered questions about the gut-brain axis in AD, despite tremendous advancements. Most research has been conducted on animal models and small-scale human studies, which has limited the conclusions. For example, regarding FMT as an AD complementary therapy, a limited number of studies have been conducted in mice/rats, with promising but not conclusive results. Regarding humans, only two case studies showing promising results have been conducted so far [25]. Because of small-sample studies, the results may not be generalizable and potentially contain confounding biases from subgroup factors like lifestyle, gender, ethnicity, and others that could also be associated with different gut mycobiome signatures [65]. To determine the causal links between the development of AD and gut microorganisms, longitudinal studies, systematic reviews, and metagenomic and Mendelian randomization studies that monitor microbiome changes over time in large would be crucial. Individuals vary remarkably in the composition of their microbiomes, which influences how they react to treatments [71]. Large-scale clinical trials are required to assess the effectiveness of individual microbiome-modulating treatments [24].

## Conclusions

This narrative review outlines the GMB participation and contribution to the characteristic progressive neurodegeneration of AD and entails the crucial role of the gut-brain axis in affiliation with neurohealth. The GMB produces various metabolites that contribute to the intestinal mucosa to maintain holistic health and homeostasis. Additionally, the research reviewed in this article highlights the prospective utilization of GMB-based therapeutics for AD treatment and/or management. Dietary factors have implications to influence the risk and even the inflammation associated with AD; specifically, deficiencies in antioxidants, which reduce Aβ-induced lipid peroxidation and oxidative stress. Furthermore, there is evidence that the MIND diet perpetuates neuroprotective effects observed in AD, including improvement in cognitive deficits, memory, and spatial learning. Finally, FMT studies conducted in animals suggested beneficial regulation of gut microbes and improvement in cognitive deficiencies. Despite the promising role of diet and GMB in enhancing cognition and overall brain health, there remains a discernment in various AD studies due to preliminary research conducted only on animal specimens or on small sample sizes. More large-scale trials should be conducted with a focus on the targets as reviewed in this narrative, particularly the microbiome-gut-brain axis, which can greatly impact the morbidity and mortality associated with AD.
